# Decoding the association between CT-derived body composition metrics and prognosis in stage II rectal cancer

**DOI:** 10.1186/s13244-026-02276-7

**Published:** 2026-04-20

**Authors:** Fengli Jiang, Ziyan Weng, Zhiqing Shao, Qianling Li, Jie Lin, Dening Ma, Xinyi Gao

**Affiliations:** 1 Department of Radiology, Zhejiang Cancer Hospital, 310022 Hangzhou, Zhejiang China; 2 Postgraduate training base Alliance of Wenzhou Medical University (Zhejiang Cancer Hospital), 310022 Hangzhou, Zhejiang Province China

**Keywords:** Body composition metrics, Stage II rectal cancer, Computed tomography, Prognosis

## Abstract

**Objectives:**

To evaluate the prognostic impact of CT-derived body composition metrics and clinical factors, and to develop a prognostic model in patients with stage II rectal cancer.

**Materials and methods:**

This retrospective study analyzed consecutive stage II rectal cancer patients who underwent radical surgery. The predictive value of body composition metrics and clinical factors was evaluated. A Cox proportional hazards model-derived nomogram, based on independent risk factors identified through univariate and multivariate analyses, was established and validated to predict overall survival (OS).

**Results:**

Among 975 patients (median age 63 years, IQR 55–70 years; 644 [66.1%] males), 183 deaths were recorded during a median follow-up period of 53 months (IQR 33–83 months). Low skeletal muscle density (SMD) (OR = 0.43; 95% CI: 0.20, 0.91), age ≥ 65 years, and CA125 positive (*p* < 0.05) were risk factors for severe postoperative complications. High visceral-to-subcutaneous adipose ratio (VSR) (OR = 1.33; 95% CI: 1.02, 1.73), body mass index (BMI) > 24.9, and T4 stage (*p* < 0.05) were risk factors for prolonged hospitalization. After univariate and multivariate analyses, the nomogram based on age, gross type, perineural invasion, lymphovascular invasion, inflammatory burden index, subcutaneous fat area (SFA), and SMD (*p* < 0.05) exhibited area under the curve values of 0.77/0.62, 0.77/0.62, and 0.75/0.67 at 1-year, 3-year, and 5-year OS in training/validation sets, respectively.

**Conclusions:**

In predicting the prognosis of stage II rectal cancer patients, VSR, SFA, and SMD were superior to other body composition metrics. The nomogram integrating body composition metrics and clinical factors showed superior predictive performance for OS compared to a single risk factor alone.

**Critical relevance statement:**

CT-derived body composition metrics can predict the prognosis of rectal cancer patients by reflecting the nutritional and metabolic status.

**Key Points:**

- Body composition metrics’ prognostic utility in stage II rectal cancer is clear.
- Body composition metrics are associated with clinical outcomes in stage II rectal cancer.
- Body composition is a predictive biomarker for stage II rectal cancer.

**Graphical Abstract:**

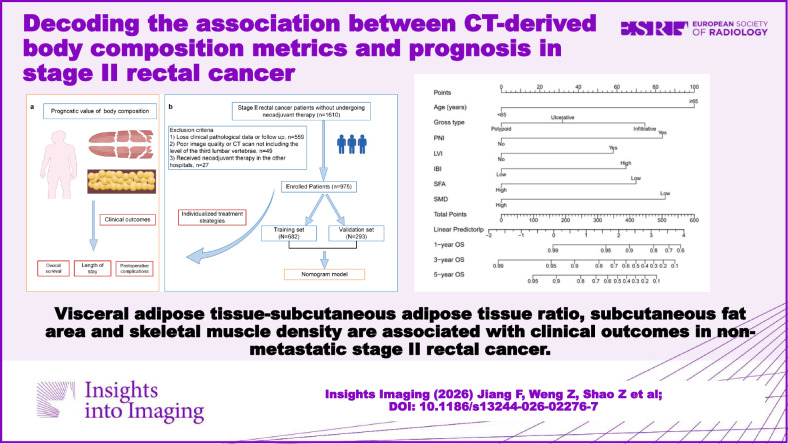

## Introduction

Colorectal cancer (CRC) is the third most common malignancy globally, imposing a heavy societal burden [1]. While stage II CRC patients have a relatively high overall survival (OS) rate, ~20%–25% experience recurrence/metastasis, leading to life-threatening risks and significant prognosis variability [2]. Thus, precise individualized prognostic prediction for stage II patients is urgently needed to guide personalized treatment and improve survival outcomes.

Cancer is increasingly viewed as a metabolic disease, with body composition emerging as a crucial predictor of treatment response and survival outcomes [3]. Traditional metrics like body mass index (BMI) fail to distinguish muscle/adipose tissue or assess their distribution [4, 5]. In contrast, CT—the gold standard for body composition assessment [6]—enables noninvasive, objective quantification of muscle and fat. This aids preoperative prognostic stratification, treatment decisions, and survival prediction in CRC.

Rectal cancer, specifically, comprises approximately 30% of CRC cases [7]. Evidence shows significant anatomical, etiological, and survival differences between colon and rectal cancer [8], so prior studies treating them as a single entity may yield ambiguous prognostic insights [9]. Thus, to boost reproducibility and generalizability, studies on homogeneous patient groups are essential.

This study aimed to investigate the prognostic significance of body composition in stage II rectal cancer patients without undergoing neoadjuvant therapy. The primary objectives were to assess the association between body composition and OS and to further integrate clinical risk factors to develop a nomogram for predicting patient prognosis. Secondary analyses investigated the correlations between body composition and postoperative complications, as well as prolonged length of stay (LOS).

## Materials and methods

### Patients

This retrospective observational study was in accordance with the Declaration of Helsinki and obtained institutional review board approval from the Ethics Committee of Zhejiang Cancer Hospital (IRB-2023-562), with written informed consent waived according to the rules.

**Fig. 1:**
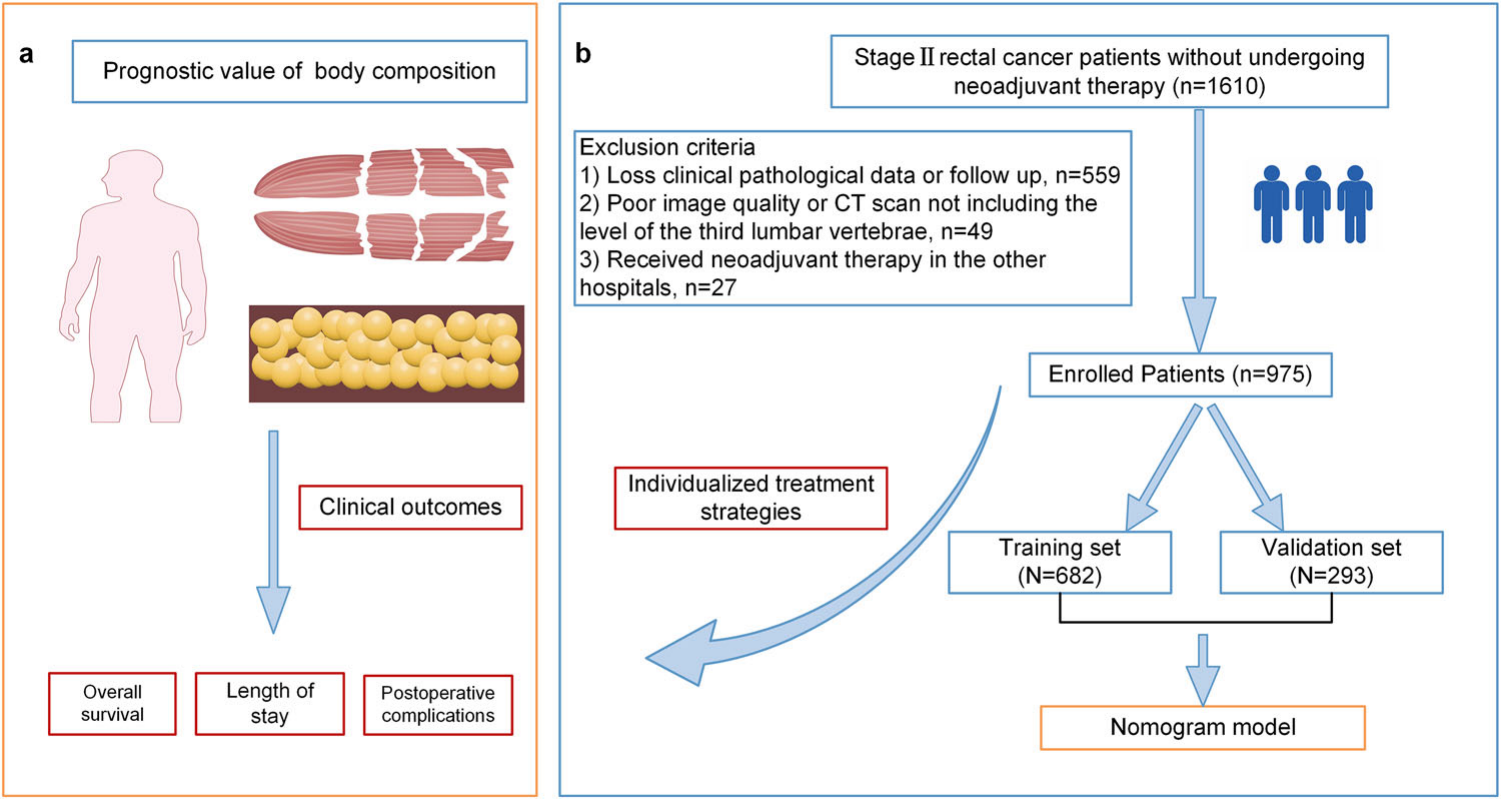
The flowchart of this study. **a** shows the research mechanism diagram of this study, **b** shows the study flowchart

Consecutive patients with stage II rectal cancer who underwent radical resection between 2010 and 2022 without undergoing neoadjuvant therapy were retrospectively reviewed. Inclusion criteria were: (1) histopathologically confirmed stage II (T3-4N0M0) primary rectal cancer according to the AJCC 8th edition staging system; (2) underwent radical surgery; and (3) had an abdominal CT scan performed within three months before surgery. Exclusion criteria included: (1) absence of complete clinical, pathological, and follow-up data; (2) poor image quality or CT scans that did not include the level of the third lumbar vertebra (L3); and (3) having received neoadjuvant therapy in the other hospitals, as this may alter body composition parameters [10]. The patient selection flowchart is shown in Fig. 1. All patients were randomly allocated into two cohorts at a ratio of 7:3.

### Data acquisition

Clinical and pathological data were extracted from the local medical record system for all patients up to March 2025, including preoperative parameters: age, sex, BMI, preoperative bowel obstruction, family history of cancer, tumor location, peritoneal reflection (rectal cancer lesion above or below the peritoneal reflection), anemia, hypoalbuminemia, albumin/globulin ratio, neutrophil-to-lymphocyte ratio, inflammatory burden index (IBI = C-reactive protein × neutrophil/lymphocyte) [11], carcinoembryonic antigen, carbohydrate antigens: CA19-9, CA125, CA72-4, and CA242. Postoperative variables included: gross type, T stage, mucinous component, tumor size, tumor number, lymph node dissection, adenoma or polyp, perineural invasion, lymphovascular invasion, schistosome, postoperative complications classified according to the Clavien-Dindo system and LOS.

Tumor size was defined as the maximum diameter of the largest axial cross-section on postoperative pathology. Postoperative complications within 30 days of surgery were graded using the Clavien-Dindo classification system [12, 13]. Complications were categorized into two groups: no/mild (grades I–II) and severe (grades III–V), with the latter defined as clinically significant events for risk stratification. LOS was computed by subtracting the surgical operation date from the first postoperative discharge date. Prolonged LOS was dichotomized at the 75th percentile (≥ 10 days vs. < 10 days) for binary outcome analysis [14].

### Image acquisition

To eliminate the confounding effects of contrast agents on muscle attenuation measurements, all imaging analyses were restricted to non-contrast CT scans [15]. See Appendix 1 (supplement) for detailed CT parameters.

### Body composition assessment

**Fig. 2:**
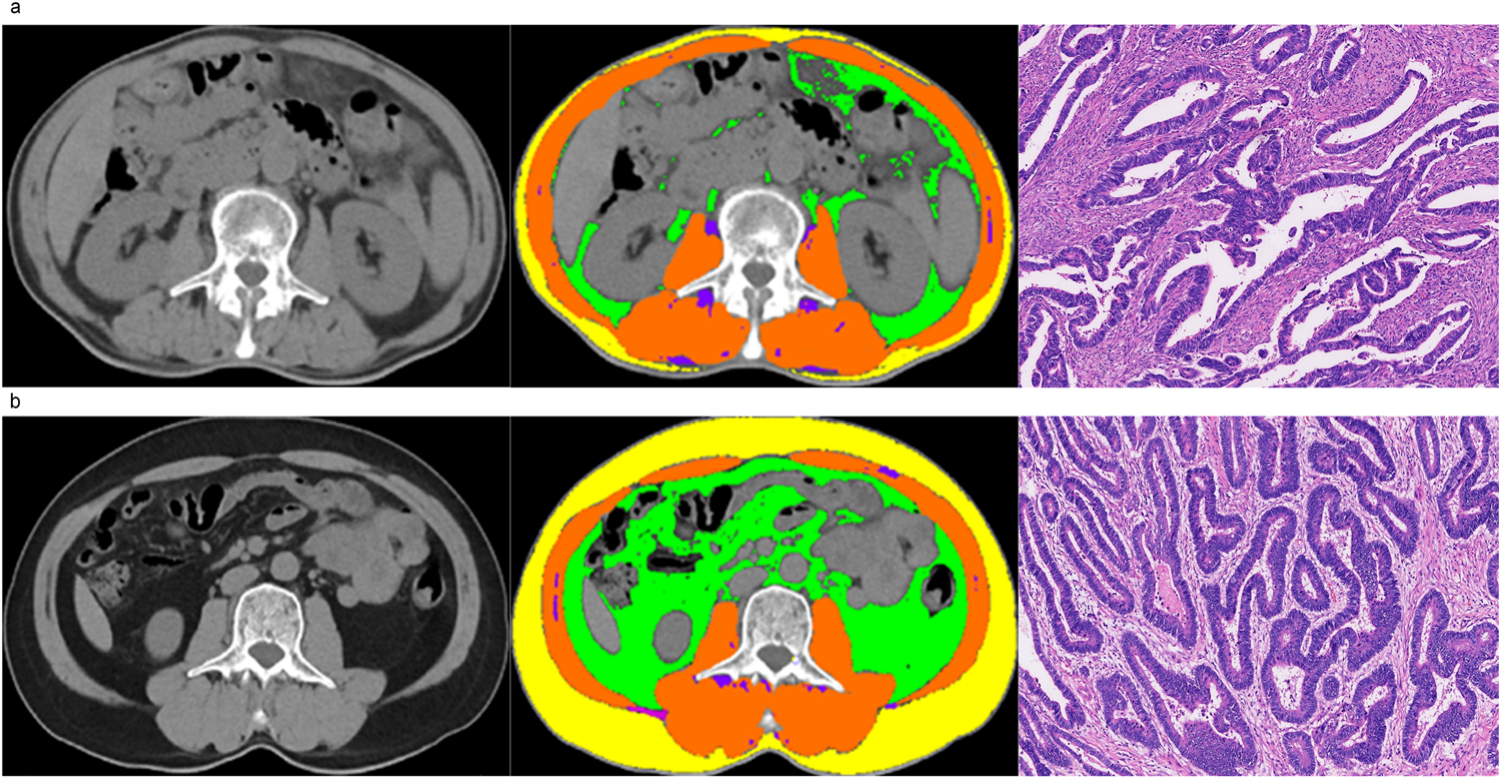
Schematic diagram of body composition evaluation at the third lumbar vertebra. The segmentation results in the middle show subcutaneous adipose tissue (SAT, yellow), visceral adipose tissue (VAT, green), skeletal muscle (SM, orange), and intermuscular adipose tissue (IMAT, purple). The rightmost part shows the Hematoxylin-Eosin-stained rectal cancer tissue (magnification, ×100). **a** Patient A: A 73-year-old female patient with stage T3N0M0 had measurements of SFA: 22.1 cm², VFA: 18.1 cm², IMFA: 8.8 cm², SMA: 107 cm², and SMD: 41.5 HU. Which means low SFA, low VFA, low IMFA, high SMA and high SMD. Her gross type was infiltrative, and she had negative perineural or lymphovascular invasion. Her overall survival was 17 months; she had no severe postoperative complications and no prolonged LOS. **b** Patient B: A 60-year-old male patient with stage T3N0M0 had measurements of SFA: 166.3 cm², VFA: 138 cm², IMFA: 6.0 cm², SMA: 138.0 cm², and SMD: 47.6 HU. Which means high SFA, low VFA, low IMFA, high SMA and high SMD. His gross type was infiltrative, and he had negative perineural invasion and positive lymphovascular invasion. His overall survival was 62 months; he had no severe postoperative complications and no prolonged LOS. SFA, subcutaneous fat area; VFA, visceral fat area; IMFA, intermuscular fat area; SMA, skeletal muscle area; SMD, skeletal muscle density; LOS, length of stay

We employed semi-automated software (SliceOmatic v5.0, Tomovision) to quantify the area and radiodensity of different body compositions at the third lumbar vertebra level [16] (Fig. 2). All CT image analyses were first performed by one trained radiologist (F.L.J., with 5 years’ experience) and then reviewed by another experienced abdominal radiologist (X.Y.G., with 10 years’ experience) following standardized procedures. Both were blinded to recipients’ clinical or pathological data and outcomes. See Appendix 2 (supplement) for body composition assessment details.

### Outcomes

The primary outcome was OS, defined as the time interval from surgical intervention to death from any cause or last follow-up. Secondary outcomes included severe postoperative complications (Clavien-Dindo grade ≥ III) and prolonged LOS (> 10 days).

### Statistical analysis

Continuous variables were summarized as mean (standard deviation, SD) or median (interquartile range, IQR), whereas categorical variables were reported as frequencies (percentage). The chi-square test was used for inter-group comparisons of categorical variables. Sex-stratified cut-off values for body composition parameters were determined using X-tile software (version 3.6.1; Yale University School of Medicine), with patients categorized into high/low groups based on optimal thresholds. The prognostic biomarkers IBI and albumin/globulin ratio underwent similar threshold determination via this methodological approach.

We employed stepwise logistic regression to evaluate associations between body composition metrics and prolonged LOS (≥ 10 days) or postoperative complications (Clavien-Dindo ≥ III). Survival analysis utilized univariable and multivariable Cox proportional-hazards models. A novel nomogram was developed by integrating clinical risk factors and body composition metrics to quantify OS associations. Model discrimination was quantified using Harrell’s concordance index (C-index) with 95% confidence intervals (CI) with optimism-corrected bootstrapping (1000 resamples). Predictive performance was evaluated through time-dependent ROC analysis (area under the curve, AUC), calibration plots, and decision curve analysis. Kaplan–Meier survival curves were generated to visualize time-to-event distributions, and the log-rank test was applied to compare survival differences between groups.

The “rms” package was utilized to generate the nomogram and calibration plots, while the “dcurves” package facilitated decision curve analysis (DCA). All statistical analyses and visualizations were executed using R 4.4.2 (R Foundation for Statistical Computing). The statistical significance level was defined as *p* < 0.05.

## Results

### Baseline characteristics

A total of 975 stage Ⅱ rectal cancer patients (644 males (66.1%) and 331 females (33.9%); median age, 63 years, IOR, 55–70 years) were included in this study. The median OS for all patients was 53 months (IQR, 33–83 months). As of last update (March 26, 2025), 183 patients have died (18.8%). The clinicopathological characteristics and multiparameter body composition of the patients in the training set are presented in Table 1.

**Table 1:** Patient’s baseline characteristics by survivor status in the training set

Characteristic	Total	Survivors	Non-survivors	*p-*value
	*N* = 682 (%)	*N* = 554 (%)	*N* = 128 (%)	
Age (years)				< 0.001*
< 65	379 (55.6)	338 (61.0)	41 (32.0)	
≥ 65	303 (44.4)	216 (39.0)	87 (68.0)	
Sex				0.13
Female	225 (33.0)	190 (34.3)	35 (27.3)	
Male	457 (67.0)	364 (65.7)	93 (72.7)	
BMI				0.09
< 18.5	42 (6.2)	29 (5.2)	13 (10.2)	
18.5–24.9	483 (70.8)	393 (70.9)	90 (70.3)	
> 24.9	157 (23.0)	132 (23.9)	25 (19.5)	
Preoperative bowel obstruction				0.58
No	620 (90.9)	502 (90.6)	118 (92.2)	
Yes	62 (9.1)	52 (9.4)	10 (7.8)	
Family history of cancer				0.63
No	610 (89.4)	494 (89.2)	116 (90.6)	
Yes	72 (10.6)	60 (10.8)	12 (9.4)	
Tumor location				0.005*
Mid-high rectum	564 (82.7)	469 (84.7)	95 (74.2)	
Low rectum	118 (17.3)	85 (15.3)	33 (25.8)	
Peritoneal reflection				0.007*
Above	530 (77.7)	442 (79.8)	88 (68.8)	
Below	152 (22.3)	112 (20.2)	40 (31.2)	
T stage				< 0.001*
T3	419 (61.4)	365 (65.9)	54 (42.2)	
T4	263 (38.6)	189 (34.1)	74 (57.8)	
Gross type				0.35
Polypoid	136 (19.9)	116 (20.9)	20 (15.6)	
Infiltrative	231 (33.9)	183 (33.1)	48 (37.5)	
Ulcerative	315 (46.2)	255 (46.0)	60 (46.9)	
Mucinous component				0.49
No	612 (89.7)	495 (89.4)	117 (91.4)	
Yes	70 (10.3)	59 (10.6)	11 (8.6)	
Tumor size				0.52
< 5	401 (58.8)	329 (59.4)	72 (56.3)	
≥ 5	281 (41.2)	225 (40.6)	56 (43.7)	
Tumor number				0.09
Single	555 (81.4)	444 (80.1)	111 (86.7)	
Multiple	127 (18.6)	110 (19.9)	17 (13.3)	
Lymph node dissection				0.09
≥ 12	632 (92.7)	518 (93.5)	114 (89.1)	
< 12	50 (7.3)	36 (6.5)	14 (10.9)	
Adenoma or polyp				0.68
No	547 (80.2)	446 (80.5)	101 (78.9)	
Yes	135 (19.8)	108 (19.5)	27 (21.1)	
Perineural invasion				0.009*
No	565 (82.8)	469 (84.7)	96 (75.0)	
Yes	117 (17.2)	85 (15.3)	32 (25.0)	
Lymphovascular invasion				0.004*
No	620 (90.9)	512 (92.4)	108 (84.4)	
Yes	62 (9.1)	42 (7.6)	20 (15.6)	
Schistosome				0.02*
No	643 (94.3)	528 (95.3)	115 (89.8)	
Yes	39 (5.7)	26 (4.7)	13 (10.2)	
Anemia				0.07
No	416 (61.0)	347 (62.6)	69 (53.9)	
Yes	266 (39.0)	207 (37.4)	59 (46.1)	
Hypoalbuminemia				0.47
No	648 (95.0)	528 (95.3)	120 (93.8)	
Yes	34 (5.0)	26 (4.7)	8 (6.2)	
*A*/*G* Ratio				< 0.001*
Low	147 (21.5)	104 (18.8)	43 (33.6)	
High	535 (78.5)	450 (81.2)	85 (66.4)	
NLR				0.22
<3	498 (73.0)	399 (72.0)	99 (77.3)	
≥3	184 (27.0)	155 (28.0)	29 (22.7)	
IBI				< 0.001*
Low	521 (76.4)	442 (79.8)	79 (61.7)	
High	161 (23.6)	112 (20.2)	49 (38.3)	
CEA				0.09
Negative	483 (70.8)	400 (72.2)	83 (64.8)	
Positive	199 (29.2)	154 (27.8)	45 (35.2)	
CA199				0.004*
Negative	628 (92.1)	518 (93.5)	110 (85.9)	
Positive	54 (7.9)	36 (6.5)	18 (14.1)	
CA125				0.11
Negative	666 (97.7)	544 (98.2)	122 (95.3)	
Positive	16 (2.3)	10 (1.8)	6 (4.7)	
CA724				0.36
Negative	602 (88.3)	486 (87.7)	116 (90.6)	
Positive	80 (11.7)	68 (12.3)	12 (9.4)	
CA242				0.75
Negative	628 (92.1)	511 (92.2)	117 (91.4)	
Positive	54 (7.9)	43 (7.8)	11 (8.6)	
SFA				< 0.001*
Low	211 (30.9)	151 (27.3)	60 (46.9)	
High	471 (69.1)	403 (72.7)	68 (53.1)	
VFA				< 0.001*
Low	386 (56.6)	294 (53.1)	92 (71.9)	
High	296 (43.4)	260 (46.9)	36 (28.1)	
IMFA				0.04*
Low	472 (69.2)	393 (70.9)	79 (61.7)	
High	210 (30.8)	161 (29.1)	49 (38.3)	
SMA				< 0.001*
Low	155 (22.7)	100 (18.1)	55 (43.0)	
High	527 (77.3)	454 (81.9)	73 (57.0)	
SMD				< 0.001*
Low	179 (26.2)	113 (20.4)	66 (51.6)	
High	503 (73.8)	441 (79.6)	62 (48.4)	
SMI				< 0.001*
Low	259 (38.0)	187 (33.8)	72 (56.3)	
High	423 (62.0)	367 (66.2)	56 (43.7)	
VSR				0.54
Low	315 (46.2)	259 (46.8)	56 (43.8)	
High	367 (53.8)	295 (53.2)	72 (56.2)	

Note: Data are numbers of patients with percentages in parentheses. Asterisk (*) was considered significant

*BMI* body mass index, *A/G* Ratio albumin/globulin ratio, *NLR* neutrophil-to-lymphocyte ratio, *IBI* inflammatory burden index, *SFA* subcutaneous fat area, *VFA* visceral fat area, *IMFA* intermuscular fat area, *SMA* skeletal muscle area, *SMD* skeletal muscle density, *SMI* skeletal muscle index, *VSR* visceral adipose tissue-subcutaneous adipose tissue ratio

The bold values with asterisk (*) were considered significant

The study included a total of 682 patients as a training set, comprising 457 males (67.0%). The internal validation set included 293 patients. There was no significant difference between the training set and the validation set (Table S1).

### Factors associated with short-term clinical outcomes

Patients with low skeletal muscle density (SMD) were at higher risk of having severe complications (Clavien-Dindo ≥ 3 grade) (OR = 0.43; 95% CI: 0.20, 0.91). In addition, age ≥ 65 years and CA125 positive were risk factors for postoperative severe complications in the logistic regression analyses (all *p* < 0.05) (Table S2).

Patients with a high visceral adipose tissue to subcutaneous adipose tissue ratio (VSR) were more likely to have a LOS of 10 days or longer (OR = 1.33; 95% CI: 1.02, 1.73). In addition, BMI > 24.9 and T4 stage were risk factors for prolonged LOS in the logistic regression analyses (all *p* < 0.05) (Table S3).

### Factors associated with long-term survival

Univariate Cox regression analysis showed that age, sex, peritoneal reflection, perineural invasion, gross type, lymphovascular invasion, schistosome, albumin/globulin ratio, IBI, CA199, and CA125 were associated with OS in the training set (all *p* < 0.05). Among body composition metrics, subcutaneous fat area (SFA), visceral fat area (VFA), intermuscular fat area (IMFA), skeletal muscle area (SMA), SMD, and SMI were also associated with OS (all *p* < 0.05, Table 2).

**Table 2:** Stepwise Cox regression analysis for overall survival in the training set

Characteristic	Univariate analysis		Multivariate analysis	
	HR (95% CI)	*p*-value	HR (95% CI)	*p*-value
Age (years)				
< 65	1		1	
≥ 65	3.60 (2.47–5.23)	< 0.001*	2.50 (1.64–3.81)	< 0.001*
Sex				
Female	1			
Male	1.60 (1.08–2.39)	0.02*		
BMI				
< 18.5	1			
18.5–24.9	0.65 (0.37–1.17)	0.15		
> 24.9	0.53 (0.27–1.04)	0.07		
Preoperative bowel obstruction				
No	1			
Yes	1.19 (0.62–2.28)	0.61		
Family history of cancer				
No	1			
Yes	0.81 (0.45–1.47)	0.49		
Tumor location				
Mid-high rectum	1			
Low rectum	1.40 (0.94–2.09)	0.09		
Peritoneal reflection				
Above	1			
Below	1.46 (1.002–2.12)	0.04*		
T stage				
T3	1			
T4	1.40 (0.98–2.01)	0.07		
Gross type				
Polypoid	1		1	
Infiltrative	1.96 (1.16–3.32)	0.012*	2.16 (1.26–3.69)	0.005*
Ulcerative	1.24 (0.75–2.06)	0.41	1.39 (0.84–2.33)	0.20
Mucinous component				
No	1			
Yes	0.87 (0.47–1.62)	0.66		
Tumor size				
< 5	1			
≥ 5	1.09 (0.77–1.55)	0.63		
Tumor number				
Single	1			
Multiple	0.93 (0.55–1.56)	0.77		
Lymph node dissection				
≥ 12	1			
< 12	1.45 (0.83–2.53)	0.19		
Adenoma or polyp				
No	1			
Yes	1.39 (0.90–2.13)	0.13		
Perineural invasion				
No	1		1	
Yes	2.06 (1.38–3.08)	< 0.001*	2.34 (1.56–3.58)	< 0.001*
Lymphovascular invasion				
No	1		1	
Yes	2.45 (1.51–3.96)	< 0.001*	1.87 (1.13–3.10)	0.02*
Schistosome				
No	1			
Yes	2.05 (1.15–3.64)	0.02*		
Anemia				
No	1			
Yes	1.26 (0.89–1.79)	0.19		
Hypoalbuminemia				
No	1			
Yes	1.93 (0.94–3.96)	0.08		
*A*/*G* Ratio				
Low	1			
High	0.53 (0.37–0.77)	< 0.001*		
NLR				
< 3	1			
≥ 3	0.99 (0.65–1.50)	0.97		
IBI				
Low	1		1	
High	2.26 (1.58–3.23)	< 0.001*	1.77 (1.21–2.58)	0.003*
CEA				
Negative	1			
Positive	1.34 (0.93–1.93)	0.12		
CA199				
Negative	1			
Positive	1.77 (1.07–2.93)	0.03*		
CA125				
Negative	1			
Positive	2.50 (1.10–5.69)	0.03*		
CA724				
Negative	1			
Positive	0.89 (0.49–1.61)	0.69		
CA242				
Negative	1			
Positive	1.16 (0.62–2.16)	0.64		
SFA				
Low	1		1	
High	0.45 (0.32–0.64)	< 0.001*	0.55 (0.37–0.80)	0.002*
VFA				
Low	1			
High	0.50 (0.34–0.73)	< 0.001*		
IMFA				
Low	1			
High	1.53 (1.07–2.19)	0.02*		
SMA				
Low	1		1	
High	0.36 (0.25–0.59)	< 0.001*	0.73 (0.48–1.11)	0.14
SMD				
Low	1		1	
High	0.31 (0.22–0.44)	< 0.001*	0.46 (0.31–0.68)	< 0.001*
SMI				
Low	1			
High	0.49 (0.35–0.70)	< 0.001*		
VSR				
Low	1			
High	1.30 (0.92–1.86)	0.14		

Note: Data in parentheses are 95% CIs. Asterisk (*) was considered significant

*BMI* body mass index, *A/G* Ratio, albumin/globulin ratio, *NLR* neutrophil-to-lymphocyte ratio, *IBI* inflammatory burden index, *SFA* subcutaneous fat area, *VFA* visceral fat area, *IMFA* intermuscular fat area, *SMA* skeletal muscle area, *SMD* skeletal muscle density, *SMI* skeletal muscle index, *VSR*, visceral adipose tissue-subcutaneous adipose tissue ratio

The bold values with asterisk (*) were considered significant

**Fig. 3:**
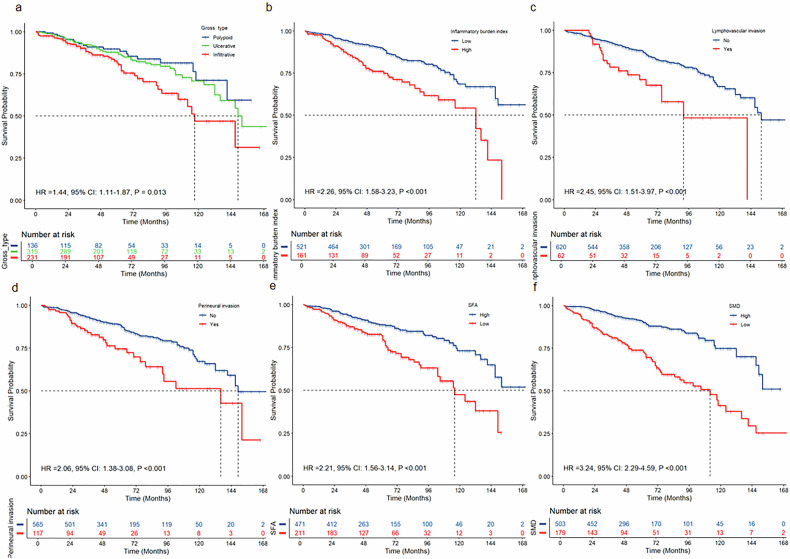
Kaplan–Meier survival curves for overall survival at the L3 level in the training set. The Kaplan–Meier survival curves for overall survival of patients grouped by gross type (**a**), inflammatory burden index (**b**), lymphovascular invasion (**c**), perineural invasion (**d**), SFA (**e**), and SMD (**f**). SFA, subcutaneous fat area; SMD, skeletal muscle density

In the multivariable analysis, age ≥ 65 (HR = 2.50; 95% CI: 1.64, 3.81; *p* < 0.001), infiltrative gross type (HR = 2.16; 95% CI: 1.26, 3.69; *p* = 0.005), with perineural invasion(HR = 2.34; 95% CI: 1.56, 3.58; *p* < 0.001), with lymphovascular invasion (HR = 1.87; 95% CI: 1.13, 3.10; *p* = 0.02) and higher IBI (HR = 1.77; 95% CI: 1.21, 2.58; *p* = 0.003) were identified as independent prognostic factors for poor OS in stage Ⅱ rectal cancer patients. While higher SFA (Male ≥ 74.79 cm², Female ≥ 63.2 cm²) (HR = 0.55; 95% CI: 0.37, 0.80; *p* = 0.002), higher SMD (Male ≥ 35.61HU, Female ≥ 33.33HU) (HR = 0.46; 95% CI: 0.31, 0.68; *p* < 0.001) were significantly associated with better OS. (Table 2 and Fig. 3; Figs. S1 and  S2).

### Nomogram model construction and evaluation for predicting long-term survival

**Fig. 4:**
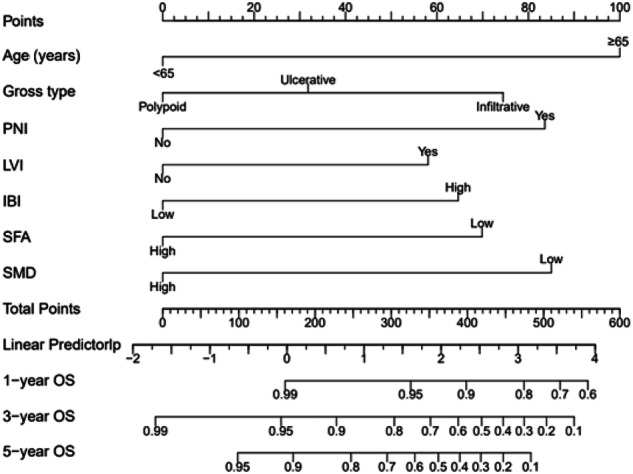
A nomogram to predict survival probability at 1, 3 and 5years in patients with stage II rectal cancer. IBI, inflammatory burden index; SFA, subcutaneous fat area; SMD, skeletal muscle density; PNI, perineural invasion; LVI, lymphovascular invasion

Multivariate Cox regression analysis identified age, gross type, perineural invasion, lymphovascular invasion, IBI, SFA, and SMD as independent predictors of postoperative OS. These variables were incorporated into a nomogram model to predict OS (Fig. 4).

**Fig. 5:**
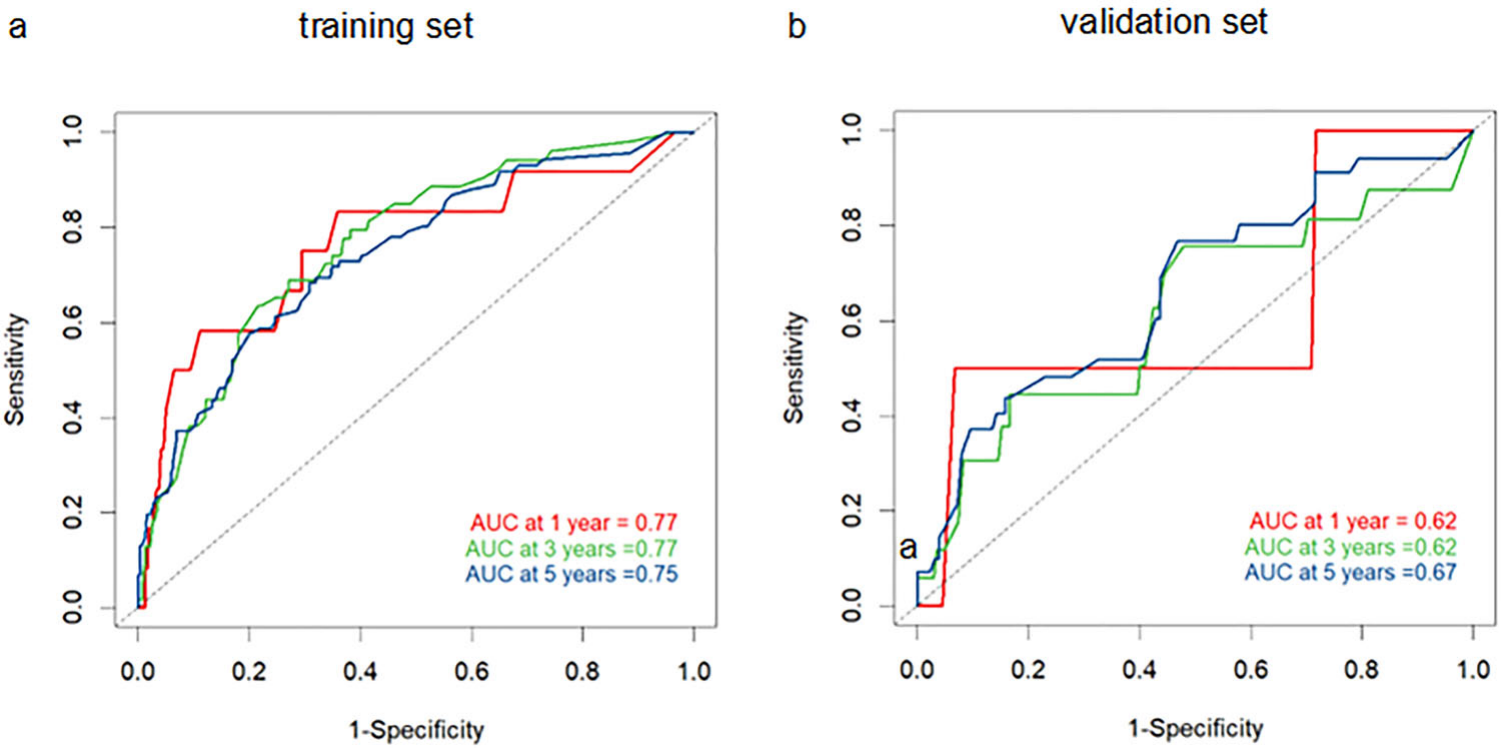
Time-ROC curves. The nomogram scores for predicting 1-year, 2-year, and 3-year high-risk groups in the training set (**a**) and validation set (**b**) were shown

Internal validation with 1000 bootstrap resamples yielded a C-index of 0.75, indicating good predictive accuracy. In the training set, the AUC of ROC curves for the nomogram at 1-year, 3-year, and 5-year were 0.77, 0.77, and 0.75, respectively (Fig. 5a). The predicted AUC values for 1-year, 3-year, and 5-year were 0.62, 0.62, and 0.67, respectively (Fig. 5b).

According to the nomogram, individualized risk scores were calculated, and patients were divided into a low-risk group (OS: risk score < 0.65) and a high-risk group (OS: risk score ≥ 0.65). Survival curves were plotted. Kaplan–Meier survival analysis showed significant differences in OS among different risk groups, indicating that the nomogram can accurately stratify the risk of stage II rectal cancer (Fig. 2 and Fig. S3).

**Fig. 6:**
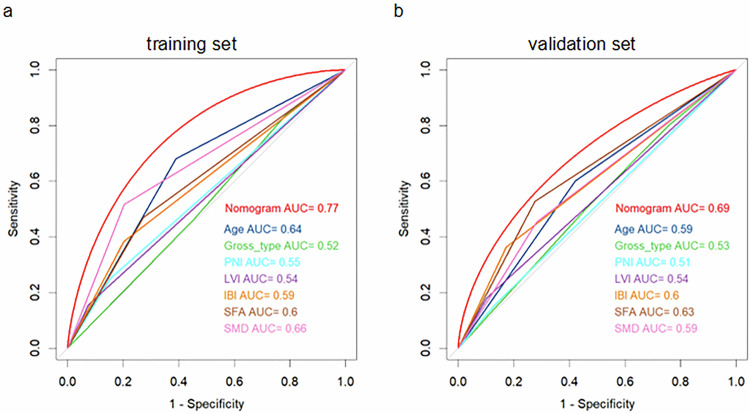
Areas under the receiver operating characteristic curves for overall survival. Index: Age, Gross type, PNI, LVI, IBI, SFA, SMD at the third lumbar level. The area under the curve of the eight indexes for overall survival in the training set (**a**) and validation set (**b**). IBI, inflammatory burden index; SFA, subcutaneous fat area; SMD, skeletal muscle density; PNI, perineural invasion; LVI, lymphovascular invasion

The nomogram outperformed individual predictors, including age, gross type, perineural or lymphovascular invasion, IBI, SFA, and SMD (Fig. 6). The calibration plot showed excellent concordance between predicted probabilities and observed outcomes (Fig. S4). DCA further confirmed the substantial clinical utility of the nomogram model (Fig. S5).

## Discussion

The objective of this study was to investigate the relationships between CT-derived body composition metrics and clinical outcomes in patients with stage II rectal cancer. Our findings indicated that low SMD was significantly associated with an increased odds of severe postoperative complications (OR = 0.43; 95% CI: 0.20, 0.91). Additionally, an elevated VSR was linked to prolonged hospitalization (OR = 1.33; 95% CI: 1.02, 1.73). We developed a nomogram that integrated preoperative body composition metrics with key clinical factors, including age, tumor gross type, perineural or lymphovascular invasion, and inflammatory burden index. This model demonstrated superior predictive capability for OS compared to individual prognostic biomarkers, making it a valuable tool for risk assessment and personalized treatment planning.

While BMI and involuntary weight loss are traditional nutritional indicators, the “obesity paradox” underscores their limitations [4, 5]. In CRC, a paradox exists: some studies link higher BMI to incidence/poor postoperative outcomes [17, 18], while others link it to better prognosis [19]. BMI fails to reflect fat distribution, muscle quality, or differentiate visceral (poor prognosis) from subcutaneous fat (protective). Consistent with prior colon cancer research [20], this study found no BMI difference between survival/death groups, but significant body composition differences—showing it overcomes BMI’s flaws.

Adipose tissue majorly includes visceral fat and subcutaneous fat [21]. Consistent with previous CRC studies [22, 23], this study found higher SFA linked to better OS, acting as an independent prognostic factor. Likely reflecting subcutaneous fat’s lower inflammation/immune cells, less metabolic syndrome involvement and protecting against cachexia/tumor progression [24]. As shown in the case depicted in Fig. 2, compared to patient B, patient A has lower subcutaneous fat, thus tending to a lower survival rate. Our model also correctly classified patients A and B into high-risk and low-risk groups. Similar results exist in post-operative NSCLC patients [6] and advanced gastric cancer patients [25]. In contrast, visceral adipocytes drive inflammation/insulin resistance by releasing adipokines and chemokines associated with metabolic syndrome, which are associated with poor tumor outcomes [26]. Huang et al found high visceral adipose tissue (VAT) linked to shorter progression-free survival in resectable locally advanced rectal cancer [27]. Interestingly, we observed a paradox: high VFA correlated with longer OS, consistent with evidence that high VAT reduces CRC lymph node metastasis (suggesting benefit/neutrality)—needs further study [28].

Prior studies have shown that higher VSR is associated with lower OS/higher mortality in rectal cancer cohorts [29, 30]. Our study showed no significant association between VSR and OS, but VSR independently predicted prolonged LOS. Similarly, Bocca G et al identified VSR as the sole independent body-composition risk factor for extended LOS [31]. These findings suggest VSR may represent a novel imaging biomarker for identifying patients at risk of adverse clinical outcomes and optimizing perioperative management strategies, e.g., nutritional support and infection prevention. Myosteatosis causes muscle metabolic dysfunction and links to increased macrophage/T cell infiltration [32]. It independently predicts poor outcomes in pancreatic [33] and biliary tract cancer [34]. SMD and IMAT are key utilized metrics to quantify muscle fat infiltration [35–37]. Xiao et al found low SMD linked to higher postoperative complication and mortality risks [38], aligning with our observation that low SMD independently predicted progression in stage II rectal cancer. This may be due to muscle mass as a key reserve of amino acids/energy substrates, essential for protein homeostasis and metabolic needs during systemic inflammation. Consistently, patients with preserved muscle mass demonstrate enhanced perioperative tolerance, as evidenced by reduced postoperative complications and accelerated recovery trajectories [39]. Conversely, Nie et al [40] and Arayne et al [41] reported no significant associations between body composition metrics and postoperative complications —discrepancies likely stem from racial differences, cut-off values, or sample sizes. We found that elevated IMFA was correlated with poorer OS, aligning with findings from prior research [23, 42].

A study including 415 patients with I-IV rectal cancer found no link between CT-derived body composition metrics and postoperative complications [40]. Conversely, a 1630-patient stage I-III CRC study found low SMI or low SMD linked to a higher risk of postoperative complications [38]. Our 975-patient stage II rectal cancer study revealed increased severe complication risks in those with low SMD. Discrepancies may stem from differences in sample size, inclusion of mixed colon/rectal cancers, and confounding pathological staging. Unlike prior studies, our cohort targets stage II rectal cancer, yielding more tailored prognostic indicators for this group.

Previous studies disagree on the optimal CT phase for body composition analysis. Shafaat et al used non-contrast CT scans (or early arterial phase if unavailable) [43]. Some studies note contrast agents may alter muscle radiation attenuation, so they included only non-contrast CT [15, 44]. We argue that intravenous contrast may introduce beam-hardening artifacts/elevate muscle attenuation, causing false-negative myosteatosis diagnoses. Thus, we used non-contrast CT for measurements (even if enhanced scans were available). However, restricting the CT phase may limit the clinical applicability of CT-based body composition assessments. Future multi-center large-sample studies should check consistency across CT phases.

This study had several strengths. Unlike prior studies, we focused specifically on stage II rectal cancer (excluding mixed colorectal cohorts). Our findings highlight the importance of muscle mass/fat storage for clinical decisions in rectal cancer, suggesting targeted nutrition/exercise interventions may improve patient care. Additionally, we integrated body composition data, the inflammatory load index, and other clinical pathological indicators into a comprehensive nomogram. This model exhibits high predictive accuracy and interpretability, ultimately contributing to improved survival outcomes for rectal cancer patients.

This study also had several limitations. Firstly, as a long-term retrospective study, advancements in imaging techniques (e.g., CT protocols) and examination methods may have led to uncaptured prognostic variables. Secondly, despite a relatively large sample size, multicenter prospective studies are needed to validate the model’s clinical applicability. Thirdly, consistent with previous studies [23, 36], a ≤ 3-month abdominal CT-surgical interval may see significant body composition changes, potentially impacting prognosis prediction accuracy. Fourthly, consistent with prior studies [20, 23], manual semi-quantitative delineation loses spatial information, highlighting the need for automated 3D image acquisition.

In conclusion, in predicting the prognosis of stage II rectal cancer patients, VSR, SFA, and SMD are superior to other body composition metrics. A nomogram integrating body composition metrics could serve as a crucial tool for developing individualized treatment strategies and enhancing patient management.

## Supplementary information

ELECTRONIC SUPPLEMENTARY MATERIAL

## Data Availability

The datasets generated or analyzed during the study are available from the corresponding author upon reasonable request.

## Abbreviations

BMI: Body mass index
IBI: Inflammatory burden index
IMFA: Intermuscular fat area
LOS: Length of stay
OS: Overall survival
SFA: Subcutaneous fat area
SMA: Skeletal muscle area
SMD: Skeletal muscle density
SMI: Skeletal muscle index
VAT: Visceral adipose tissue
VFA: Visceral fat area
VSR: Visceral adipose tissue-subcutaneous adipose tissue ratio
