# Supplementary material for: Decoding the association between CT-derived body composition metrics and prognosis in stage II rectal cancer
Source: Insights Imaging. 2026 Apr 20;17:104. doi: 10.1186/s13244-026-02276-7 (PMC13096300; doi:10.1186/s13244-026-02276-7)
Supplement: Supplementary file 1 — ELECTRONIC SUPPLEMENTARY MATERIAL [file 13244_2026_2276_MOESM1_ESM.pdf]

Decoding the association between CT-derived body composition metrics and prognosis in stage II rectal cancer

ELECTRONIC SUPPLEMENTARY MATERIAL

Appendix 1 CT parameters.

Part of abdominal CT images were scanned by one of these CT scanners (Somatom Definition Flash, Siemens; optima CT 680 Series, GE Medical Systems; Ingenuity CT, Philips) with standardized parameters. Key technical specifications included: tube voltage (120 kV), automatic tube current modulation, matrix size (512 × 512 pixels), spacing (5 mm) and slice thickness (5 mm). Other abdominal CT images were acquired by one of these CT scanners (Sensation 16, Siemens; BrightSpeed, GE Medical Systems). The CT protocols were as follows: tube voltage (120 kV), automatic tube current modulation, matrix size (512 × 512 pixels), spacing (7 mm) and slice thickness (7 mm).

Appendix 2

Hounsfield unit (HU) thresholds were defined as follows: skeletal muscle area (SMA: −29 to +150 HU), visceral fat area (VFA: −150 to −50 HU), subcutaneous fat area (SFA: −190 to −30 HU), and intermuscular fat area (IMFA: −190 to −30 HU) [1]. Skeletal muscle density (SMD) was automatically computed by the software as the mean CT attenuation within the region of interest. The skeletal muscle index was derived by dividing total muscle cross-sectional area (cm^2) by height squared (m^2). The Visceral adipose tissue to subcutaneous adipose tissue ratio (VSR) was derived as VFA divided by SFA. Considering sex differences in body composition distribution, the optimal thresholds for body composition indices were independently established for males and females. Patients were then categorized accordingly. The specific cutoff values for each category are summarized as follows: SFA, 74.79 cm^2 for male and 63.2 cm^2 for female; VFA, 140.2 cm^2 for male and 37.9 cm^2 for female; IMFA, 18.41 cm^2 for male and 11.21 cm^2 for female; SMA, 113.4 cm^2 for male and 70.51 cm^2 for female; SMD, 35.61 HU for male and 30.33 HU for female; the skeletal muscle index, 42.64 for male and 35.64 for female; VSR, 0.78 for male and 0.82 for female.

Supplement References:

1. Cespedes Feliciano EM, Popuri K, Cobzas D, et al (2020) Evaluation of automated computed tomography segmentation to assess body composition and mortality associations in cancer patients. J Cachexia Sarcopenia Muscle 11(5):1258-1269.

Table S1 Patients' baseline characteristics between training and validation sets

Characteristic	Total	Training set	Validation set	*p*-value
	N= 975(%)	N=682(%)	N= 293(%)	
Age (years)				0.83
<65	544 (55.8)	379 (55.6)	165 (56.3)	
≥65	431 (44.2)	303 (44.4)	128 (43.7)	
Sex				0.34
Female	331 (33.9)	225 (33.0)	106 (36.2)	
Male	644 (66.1)	457 (67.0)	187 (63.8)	
BMI				0.59
<18.5	63 (6.5)	42 (6.2)	21 (7.2)	
18.5-24.9	681 (69.8)	483 (70.8)	198 (67.6)	
>24.9	231 (23.7)	157 (23.0)	74 (25.2)	
Preoperative bowel obstruction				0.82
No	885 (90.8)	620 (90.9)	265 (90.4)	
Yes	90 (9.2)	62 (9.1)	28 (9.6)	
Family history of cancer				0.35
No	866 (88.8)	610 (89.4)	256 (87.4)	
Yes	109 (11.2)	72 (10.6)	37 (12.6)	
Tumor location				0.5
Mid-high rectum	811 (83.2)	564 (82.7)	247 (84.3)	
Low rectum	164 (16.8)	118 (17.3)	46 (15.7)	
Peritoneal reflection				0.46
Above	764 (78.4)	530 (77.7)	234 (79.9)	
Below	211 (21.6)	152 (22.3)	59 (20.1)	
T stage				0.76
T3	596 (61.1)	419 (61.4)	177 (60.4)	
T4	379 (38.9)	263 (38.6)	116 (39.6)	
Gross type				0.10
Polypoid	185 (19.0)	136 (19.9)	49 (16.7)	
Infiltrative	318 (32.6)	231 (33.9)	87 (29.7)	
Ulcerative	472 (48.4)	315 (46.2)	157 (53.6)	
Mucinous component				0.24
No	882 (90.5)	612 (89.7)	270 (92.1)	
Yes	93 (9.5)	70 (10.3)	23 (7.9)	
Tumor size				0.86
<5	575 (59.0)	401 (58.8)	174 (59.4)	
≥5	400 (41.0)	281 (41.2)	119 (40.6)	
Tumor number				0.08
Single	779 (79.9)	555 (81.4)	224 (76.5)	
Multiple	196 (20.1)	127 (18.6)	69 (23.5)	
Lymph node dissection				0.24
≥12	897 (92.0)	632 (92.7)	265 (90.4)	

Characteristic	Total	Training set	Validation set	*p*-value
	N= 975(%)	N=682(%)	N= 293(%)	
<12	78 (8.0)	50 (7.3)	28 (9.6)	
Adenoma or polyp				0.10
No	795 (81.5)	547 (80.2)	248 (84.6)	
Yes	180 (18.5)	135 (19.8)	45 (15.4)	
Perineural invasion				0.77
No	810 (83.1)	565 (82.8)	245 (83.6)	
Yes	165 (16.9)	117 (17.2)	48 (16.4)	
Lymphovascular invasion				0.91
No	887 (91.0)	620 (90.9)	267 (91.1)	
Yes	88 (9.0)	62 (9.1)	26 (8.9)	
Schistosome				0.20
No	925 (94.9)	643 (94.3)	282 (96.2)	
Yes	50 (5.1)	39 (5.7)	11 (3.8)	
Anemia				0.94
No	594 (60.9)	416 (61.0)	178 (60.8)	
Yes	381 (39.1)	266 (39.0)	115 (39.2)	
Hypoalbuminemia				0.40
No	930 (95.4)	648 (95.0)	282 (96.2)	
Yes	45 (4.6)	34 (5.0)	11 (3.8)	
A/G Ratio				0.57
Low	215 (22.1)	147 (21.6)	68 (23.2)	
High	760 (77.9)	535 (78.4)	225 (76.8)	
NLR				0.26
<3	722 (74.1)	498 (73.1)	224 (76.5)	
≥3	253 (25.9)	184 (26.9)	69 (23.5)	
IBI				0.15
Low	757 (77.6)	521 (76.4)	236 (80.5)	
High	218 (22.4)	161 (23.6)	57 (19.5)	
CEA				0.31
Negative	681 (69.8)	483 (70.8)	198 (67.6)	
Positive	294 (30.2)	199 (29.2)	95 (32.4)	
CA199				0.89
Negative	897 (92.0)	628 (92.1)	269 (91.8)	
Positive	78 (8.0)	54 (7.9)	24 (8.2)	
CA125				0.53
Negative	954 (97.8)	666 (97.7)	288 (98.3)	
Positive	21 (2.2)	16 (2.3)	5 (1.7)	
CA724				0.19
Negative	869 (89.1)	602 (88.3)	267 (91.1)	
Positive	106 (10.9)	80 (11.7)	26 (8.9)	
CA242				0.83

Characteristic	Total	Training set	Validation set	*p*-value
	N= 975(%)	N=682(%)	N= 293(%)	
Negative	899 (92.2)	628 (92.1)	271 (92.5)	
Positive	76 (7.8)	54 (7.9)	22 (7.5)	
SFA				0.36
Low	293 (30.1)	211 (30.9)	82 (28.0)	
High	682 (69.9)	471 (69.1)	211 (72.0)	
VFA				0.17
Low	538 (55.2)	386 (56.6)	152 (51.9)	
High	437 (44.8)	296 (43.4)	141 (48.1)	
IMFA				0.81
Low	677 (69.4)	472 (69.2)	205 (70.0)	
High	298 (30.6)	210 (30.8)	88 (30.0)	
SMA				0.11
Low	208 (21.3)	155 (22.7)	53 (18.1)	
High	767 (78.7)	527 (77.3)	240 (81.9)	
SMD				0.51
Low	250 (25.6)	179 (26.2)	71 (24.2)	
High	725 (74.4)	503 (73.8)	222 (75.8)	
SMI				0.89
Low	369 (37.8)	259 (38.0)	110 (37.5)	
High	606 (62.2)	423 (62.0)	183 (62.5)	
VSR				0.60
Low	445 (45.6)	315 (46.2)	130 (44.4)	
High	530 (54.4)	367 (53.8)	163 (55.6)	

Note: Data are numbers of patients with percentages in parentheses.

Abbreviations: BMI, body mass index; A/G Ratio, albumin/globulin ratio; NLR, neutrophil-to-lymphocyte ratio; IBI, inflammatory burden index; SFA, subcutaneous fat area; VFA, visceral fat area; IMFA, intermuscular fat area; SMA, skeletal muscle area; SMD, skeletal muscle density; SMI, skeletal muscle index; VSR, visceral adipose tissue-subcutaneous adipose tissue ratio.

Table S2 Stepwise logistic regression analysis for postoperative complications

Characteristic	Univariate analysis		Multivariate analysis	
	OR (95% CI)	*p*-value	OR (95% CI)	*p*-value
Age (years)				
<65	1		1	
≥65	3.29 (1.56 ~ 6.92)	**0.002***	2.32 (1.04 ~ 5.18)	**0.04***
Sex				
Female	1			
Male	1.77 (0.79 ~ 3.93)	0.16		
BMI				
<18.5	1			
18.5-24.9	2.56 (0.34 ~ 19.15)	0.36		
>24.9	1.94 (0.23 ~ 16.04)	0.54		
Preoperative bowel obstruction				
No	1			
Yes	1.68 (0.63 ~ 4.43)	0.29		
Family history of cancer				
No	1			
Yes	1.03 (0.36 ~ 2.96)	0.96		
Tumor location				
Mid-high rectum	1			
Low rectum	1.02 (0.42 ~ 2.51)	0.96		
Peritoneal reflection				
Above	1			
Below	0.74 (0.30 ~ 1.81)	0.51		
T stage				
T3	1			
T4	1.05 (0.53 ~ 2.09)	0.89		
Gross type				
Polypoid	1			
Infiltrative	0.97 (0.346 ~ 2.71)	0.95		
Ulcerative	1.25 (0.49 ~ 3.18)	0.64		
Mucinous component				
No	1			
Yes	1.61 (0.61 ~ 4.27)	0.34		
Tumor size				
<5	1			
≥5	1.37 (0.70 ~ 2.70)	0.36		
Tumor number				
Single	1			
Multiple	1.87 (0.90 ~ 3.89)	0.09		

Lymph node dissection				
≥12	1			
<12	1.51 (0.52 ~ 4.39)	0.45		
Adenoma or polyp				
No	1			
Yes	0.73 (0.279 ~ 1.90)	0.52		
Perineural invasion				
No	1			
Yes	1.02 (0.42 ~ 2.49)	0.97		
Lymphovascular invasion				
No	1			
Yes	0.94 (0.28 ~ 3.14)	0.92		
Schistosome				
No	1			
Yes	2.59 (0.85 ~ 7.40)	0.09		
Anemia				
No	1			
Yes	1.18 (0.60 ~ 2.33)	0.64		
Hypoalbuminemia				
No	1			
Yes	3.75 (1.38 ~ 10.18)	**0.009***		
A/G Ratio				
Low	1			
High	0.53 (0.26 ~ 1.08)	0.08		
NLR				
<3	1			
≥3	1.72 (0.86 ~ 3.48)	0.13		
IBI				
Low	1			
High	2.12 (1.05 ~ 4.28)	**0.04***		
CEA				
Negative	1			
Positive	1.28 (0.60 ~ 2.48)	0.59		
CA199				
Negative	1			
Positive	1.51 (0.52 ~ 4.39)	0.45		
CA125				
Negative	1		1	
Positive	9.63 (3.31 ~ 28.00)	**<0.001***	6.73 (2.22 ~ 20.44)	**<0.001***
CA724				
Negative	1			
Positive	1.738 (0.70 ~ 4.29)	0.23		

CA242				
Negative	1			
Positive	2.04 (0.77 ~ 5.42)	0.15		
SFA				
Low	1			
High	0.63 (0.32 ~ 1.26)	0.19		
VFA				
Low	1			
High	0.72 (0.36 ~ 1.45)	0.35		
IMFA				
Low	1			
High	1.54 (0.77 ~ 3.07)	0.22		
SMA				
Low	1			
High	0.39 (0.20 ~ 0.78)	**0.008***		
SMD				
Low	1		1	
High	0.27 (0.14 ~ 0.54)	**<0.001***	0.43 (0.20 ~ 0.91)	**0.03***
SMI				
Low	1			
High	0.56 (0.29 ~ 1.11)	0.09		
VSR				
Low	1			
High	1.87 (0.91 ~ 3.86)	0.09		

Note: Data in parentheses are 95% confidence intervals (CI). Asterisk (*) was considered significant.

Abbreviations: BMI, body mass index; A/G Ratio, albumin/globulin ratio; NLR, neutrophil-to-lymphocyte ratio; IBI, inflammatory burden index; SFA, subcutaneous fat area; VFA, visceral fat area; IMFA, intermuscular fat area; SMA, skeletal muscle area; SMD, skeletal muscle density; SMI, skeletal muscle index; VSR, visceral adipose tissue-subcutaneous adipose tissue ratio.

Table S3 Stepwise logistic regression analysis for prolonged length of stay

Characteristic	Univariate analysis		Multivariate analysis	
	OR (95% CI)	*p*-value	OR (95% CI)	*p*-value
Age (years)				
<65	1			
≥65	0.98 (0.76 ~ 1.27)	0.88		
Sex				
Female	1			
Male	1.03 (0.79 ~ 1.35)	0.82		
BMI				
<18.5	1		1	
18.5-24.9	1.90 (1.12 ~ 3.21)	**0.02***	1.69 (0.99 ~ 2.89)	0.06
>24.9	2.26 (1.28 ~ 3.99)	**0.005***	1.87 (1.03 ~ 3.37)	**0.04***
Preoperative bowel obstruction				
No	1			
Yes	1.11 (0.72 ~ 1.72)	0.64		
Family history of cancer				
No	1			
Yes	1.32 (0.88 ~ 1.99)	0.18		
Tumor location				
Mid-high rectum	1			
Low rectum	1.23 (0.88 ~ 1.74)	0.23		
Peritoneal reflection				
Above	1			
Below	1.25 (0.92 ~ 1.70)	0.16		
T stage				
T3	1		1	
T4	1.32 (1.02 ~ 1.71)	**0.04***	1.32 (1.02 ~ 1.72)	**0.04***
Gross type				
Polypoid	1			
Infiltrative	1.04 (0.73 ~ 1.50)	0.82		
Ulcerative	1.26 (0.89 ~ 1.77)	0.19		

Mucinous component		
No	1	
Yes	1.44 (0.93 ~ 2.24)	0.11
Tumor size		
<5	1	
≥5	1.19 (0.92 ~ 1.54)	0.19
Tumor number		
Single	1	
Multiple	0.86 (0.63 ~ 1.18)	0.36
Lymph node dissection		
≥12	1	
<12	1.04 (0.65 ~ 1.66)	0.86
Adenoma or polyp		
No	1	
Yes	1.15 (0.83 ~ 1.60)	0.39
Perineural invasion		
No	1	
Yes	0.83 (0.59 ~ 1.15)	0.26
Lymphovascular invasion		
No	1	
Yes	1.31 (0.83 ~ 2.04)	0.25
Schistosome		
No	1	
Yes	0.86 (0.49 ~ 1.53)	0.61
Anemia		
No	1	
Yes	1.20 (0.93 ~ 1.56)	0.16
Hypoalbuminemia		
No	1	
Yes	1.003 (0.55 ~ 1.83)	0.99
A/G Ratio		
Low	1	
High	0.96 (0.71 ~ 1.30)	0.79
NLR		
<3	1	
≥3	1.11 (0.83 ~ 1.48)	0.49
IBI		
Low	1	
High	1.21 (0.89 ~ 1.65)	0.21
CEA		
Negative	1	

Positive	1.04 (0.79 ~ 1.37)	0.79		
CA199				
Negative	1			
Positive	1.17 (0.73 ~ 1.87)	0.52		
CA125				
Negative	1			
Positive	1.07 (0.45 ~ 2.57)	0.88		
CA724				
Negative	1			
Positive	1.05 (0.70 ~ 1.58)	0.81		
CA242				
Negative	1			
Positive	1.33 (0.82 ~ 2.15)	0.25		
SFA				
Low	1			
High	1.28 (0.97 ~ 1.67)	0.08		
VFA				
Low	1			
High	1.10 (0.85 ~ 1.42)	0.47		
IMFA				
Low	1			
High	1.12 (0.85 ~ 1.47)	0.43		
SMA				
Low	1			
High	1.06 (0.78 ~ 1.44)	0.70		
SMD				
Low	1			
High	0.87 (0.65 ~ 1.17)	0.35		
SMI				
Low	1			
High	1.013 (0.78 ~ 1.32)	0.92		
VSR				
Low	1		1	
High	1.40 (1.08 ~ 1.80)	**0.01***	1.33 (1.02 ~ 1.73)	**0.04***

Note: Data in parentheses are 95% CIs. Asterisk (*) was considered significant.

Abbreviations: BMI, body mass index; A/G Ratio, albumin/globulin ratio; NLR, neutrophil-to-lymphocyte ratio; IBI, inflammatory burden index; SFA, subcutaneous fat area; VFA, visceral fat area; IMFA, intermuscular fat area; SMA, skeletal muscle area; SMD, skeletal

muscle density; SMI, skeletal muscle index; VSR, visceral adipose tissue-subcutaneous adipose tissue ratio.

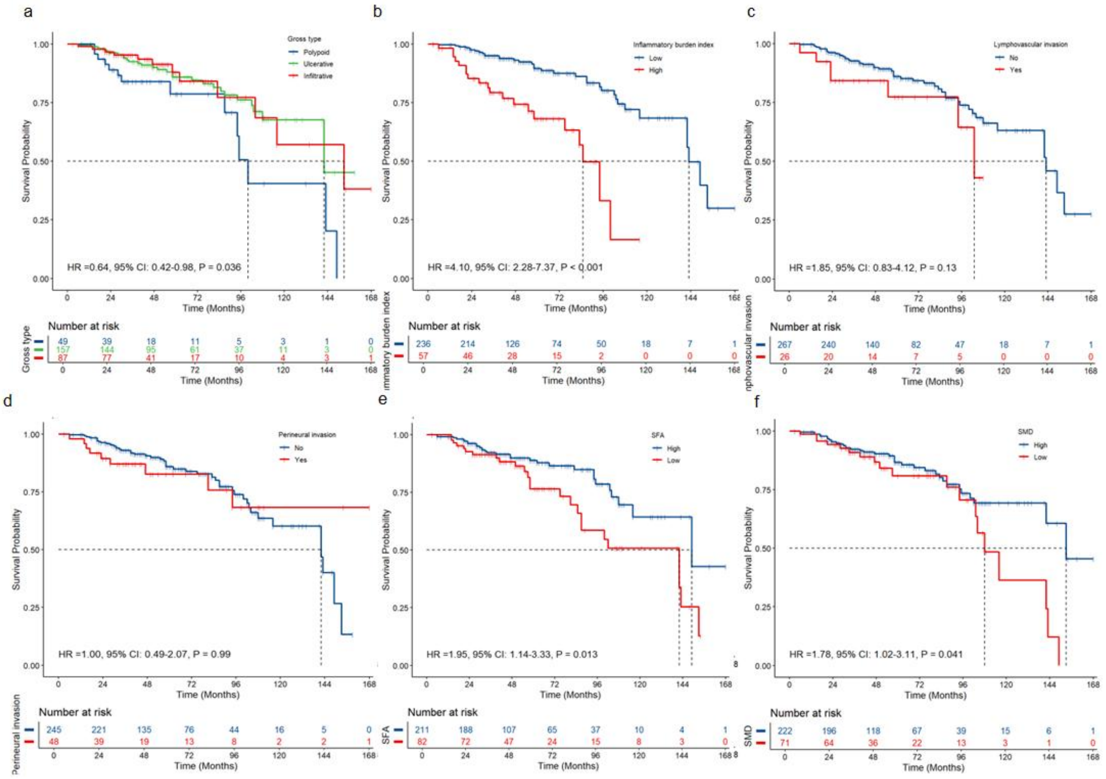

Fig. S1. Kaplan–Meier survival curves for overall survival at the L3 level in the validation set. The Kaplan-Meier survival curves for overall survival of patients grouped by gross type (a), inflammatory burden index (b), lymphovascular invasion (c), perineural invasion (d), SFA (e), and SMD (f). SFA= subcutaneous fat area, SMD= skeletal muscle density.

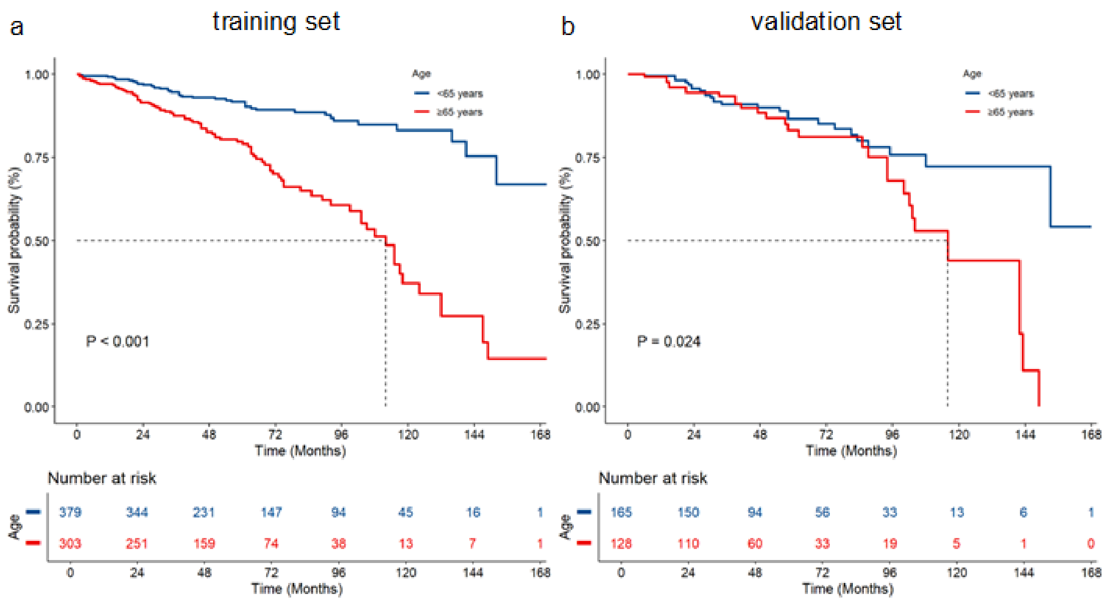

Fig. S2. Kaplan–Meier survival curves for overall survival at the L3 level. The Kaplan-Meier survival curves for overall survival of patients grouped by age in the training set (a) and validation set (b).

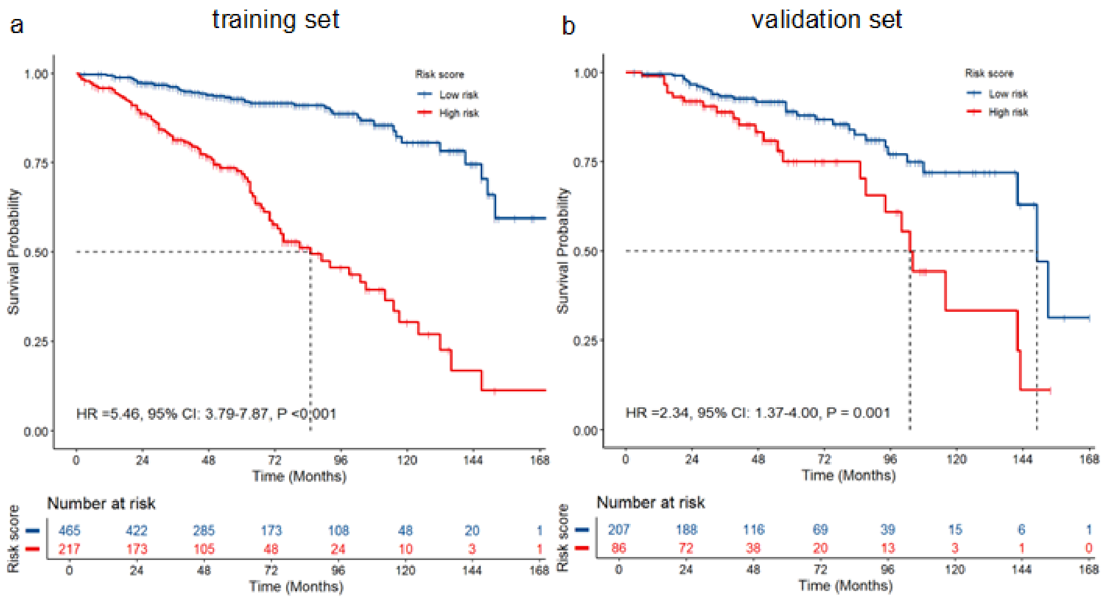

Fig. S3. Kaplan Meier curves of OS for high-risk and low-risk patients in the training (a) and validation sets (b).

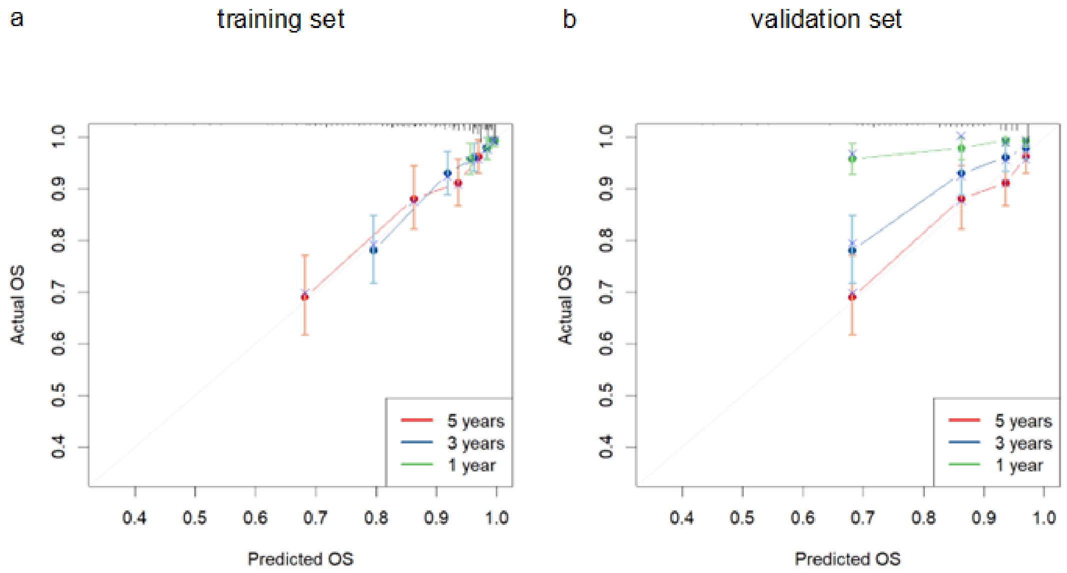

Fig. S4. Calibration curves. The nomogram in the training set (a) and validation set (b) were shown. The x-axis represents the predicted rectal cancer survival probabilities based on the prediction model, while the y-axis represents the actual outcomes from the follow-up. The diagonal gray line represented the perfect prediction of an ideal model. The solid lines represented the performance of the nomogram.

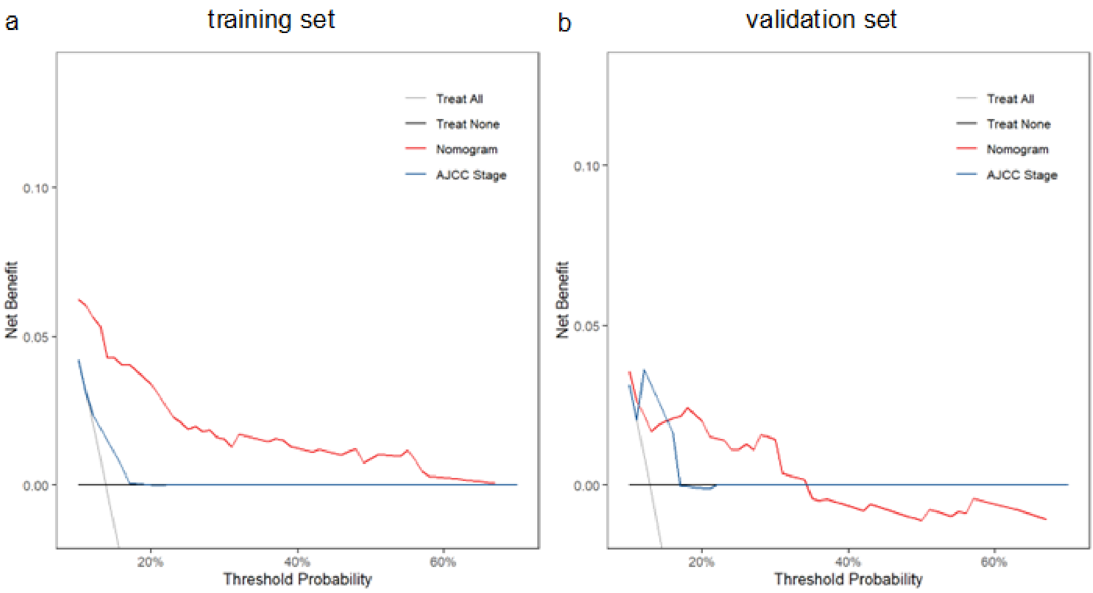

Fig. S5. The DCA of the nomogram in the training set (a) and validation set (b).
